# Medical residency: factors relating to “difficulty in helping” in the resident physician-patient relationship

**DOI:** 10.1590/S1516-31802011000100002

**Published:** 2011-01-06

**Authors:** Mario Alfredo De Marco, Vanessa Albuquerque Cítero, Maria Cezira Fantini Nogueira-Martins, Latife Yazigi, Lawrence Sagin Wissow, Luiz Antonio Nogueira-Martins, Sergio Baxter Andreoli

**Affiliations:** I PhD. Associate professor in the Department of Psychiatry, Universidade Federal de São Paulo (Unifesp), São Paulo, Brazil.; II PhD. Affiliated professor in the Department of Psychiatry, Universidade Federal de São Paulo (Unifesp), São Paulo, Brazil.; III PhD. Scientific researcher, Health Institute, Health Department of the State of São Paulo, São Paulo, São Paulo, Brazil.; IV PhD. Titular professor of the Department of Psychiatry, Universidade Federal de São Paulo (Unifesp), São Paulo, Brazil.; V PhD. Professor in the Department of Health Policy and Management, Johns Hopkins Bloomberg School of Public Health, Baltimore, United States of America.; VI PhD. Associate professor in the Department of Psychiatry, Universidade Federal de São Paulo (Unifesp), São Paulo, Brazil.

**Keywords:** Internship and residency, Physician-patient relations, Anxiety, Depression, Education, medical, Internato e residência, Relações médico-paciente, Ansiedade, Depressão, Educação médica

## Abstract

**CONTEXT AND OBJECTIVE:**

Previous studies have attempted to understand what leads physicians to label patients as ‘difficult’. Understanding this process is particularly important for resident physicians, who are developing attitudes that may have long-term impact on their interactions with patients. The aim of this study was to distinguish between patients' self-rated emotional state (anxiety and depression) and residents' perceptions of that state as a predictor of patients being considered difficult.

**DESIGN AND SETTING:**

Cross-sectional survey conducted in the hospital of Universidade Federal de São Paulo (Unifesp).

**METHODS:**

The residents completed a sociodemographic questionnaire and rated their patients using the Hospital Anxiety and Depression Scale (HADS) and Difficulty in Helping the Patient Questionnaire (DTH). The patients completed HADS independently and were rated using the Karnofsky Performance Status scale.

**RESULTS:**

On average, the residents rated the patients as presenting little difficulty. The residents' ratings of difficulty presented an association with their ratings for patient depression (r = 0.35, P = 0.03) and anxiety (r = 0.46, P = 0.02), but not with patients' self-ratings for depression and anxiety. Residents from distant cities were more likely to rate patients as difficult to help than were residents from the city of the hospital (mean score of 1.93 versus 1.07; P = 0.04).

**CONCLUSIONS:**

Understanding what leads residents to label patients as having depression and anxiety problems may be a productive approach towards reducing perceived difficulty. Residents from distant cities may be more likely to find their patients difficult.

## RESUMO

**CONTEXTO E OBJETIVO:**

Estudos têm tentado compreender o que leva os médicos a rotularem pacientes como “difíceis”. Entender este processo é particularmente importante para os médicos residentes, que estão desenvolvendo atitudes que podem ter impacto a longo prazo em suas interações com pacientes. O objetivo deste estudo foi de distinguir entre o estado emocional (ansiedade e depressão) auto-avaliado pelos pacientes e a percepção dos residentes desse estado, como preditor de pacientes serem considerados difíceis.

**TIPO DE ESTUDO E LOCAL:**

Estudo transversal realizado no hospital da Universidade Federal de São Paulo (Unifesp).

**MÉTODOS:**

Os residentes responderam a um questionário sociodemográfico e pontuaram seus pacientes com a Hospital Anxiety and Depression Scale (HADS) e o Difficulty in Helping the Patient Questionnaire (DTH). Os pacientes completaram a HADS de forma independente e foram avaliados usando o Karnofsky Performance Status Scale.

**RESULTADOS:**

Em média, os residentes avaliaram seus pacientes como mobilizadores de pouca dificuldade. Os escores de dificuldade dos residentes apresentaram associação com os escores de depressão (r = 0.35, P = 0,03) e ansiedade (r = 0,46, P = 0,02) que atribuíram aos pacientes, mas não com os escores de ansiedade e depressão na auto-avaliação dos pacientes. Residentes provenientes de cidades distantes mostraram-se mais propensos a classificar os pacientes como difíceis de ajudar do que os residentes provenientes da mesma cidade do hospital (pontuação média de 1.93 *versus* 1.07, P = 0,04).

**CONCLUSÕES:**

Compreender o que leva os residentes a classificar pacientes como tendo problemas de ansiedade e depressão pode ser uma abordagem produtiva para reduzir a dificuldade percebida. Residentes de cidades distantes do local do hospital podem ser mais propensos a considerar seus pacientes como difíceis.

## INTRODUCTION

For many generalist physicians, patients with emotional problems represent a subset of those labeled as “difficult”.^1,2^ Studies in Brazil and elsewhere in the world have found that generalists feel that they have insufficient knowledge of mental health diagnosis and treatment.^3,4^ They also worry that attempting to help patients who have mental health problems will use up scarce time or add to their own mental distress.^5^

Categorizing patients as “difficult” also implies that physicians consider that interacting with them is more stressful than is either expected or acceptable. Hahn and Hahn et al.^2,6^ estimated that this occurred in 10% to 20% of consultations in general adult outpatient settings. Physicians may respond to their discomfort in a variety of ways that are harmful to the patient-doctor relationship, including actively avoiding discussion of psychosocial issues, proposing additional medical interventions and making remarks that challenge the legitimacy of the patients' concerns.^7,8^

Understanding what makes patients “difficult” is particularly important for resident physicians who are at an early stage in their clinical training. Residents are not only developing their clinical observation and interaction skills but are also forming attitudes that will influence their future medical specialization. Several aspects of residency training may further contribute towards residents' discomfort with patients' emotional problems.^9,10^ Residents may already feel anxious about their ability to successfully care for ill patients.^10,11^ Cultural differences between trainees and patients (who in teaching hospitals often come from minority and disadvantaged social groups that are very distinct from the backgrounds of the residents) may lead to misunderstanding of patients' expressed emotional needs.^11,12^

## OBJECTIVE

Prior work has suggested that feelings of difficulty can be driven as much or more by physicians' perceptions of patients than by the patients' characteristics themselves.^12-16^ Thus, in our study, we aimed to investigate the phenomenon of “difficulty in helping” to determine whether it is associated with objectively determined patient characteristics or with physicians' perceptions or characteristics. We hypothesized that physicians would have greater difficulty in helping patients who present disabilities and psychiatric symptoms (anxiety and depressive symptoms). Our alternative hypothesis was that physicians would find patients difficult if they perceived these individuals as having psychiatric comorbidities, but that these patients would not necessarily be depressed or anxious.

## METHODS

### Design, setting and ethics

This was a cross-sectional survey of patients and resident physicians.

The study was conducted in the hospital of Universidade Federal de São Paulo (Unifesp), a major regional center for medical training and clinical care. The hospital cares for patients covered by Brazil's national health system, and they are treated by resident physicians under the direction of university teaching staff members.

The study was approved by the Research Ethics Committee of Unifesp. All participants gave their written, informed consent.

### Participants

A sample of 69 beds was created by randomly sampling the 667 national health system beds in the following wards: gastric surgery, orthopedics, internal medicine, vascular surgery, cardiology, cardiovascular surgery, neurosurgery and intensive care. For the purposes of the study, each bed represented a set made up of the patient occupying it on the day of the sampling, a family member, a resident physician and a nurse. In the present paper, we only considered the information reported by patients and residents; other results and a detailed description of the methods have been published previously.^17,18^

Beds were included if the patients occupying them were 18 years of age or older, had been hospitalized for a minimum of 24 hours and had the ability to respond to the study questionnaires. Residents were included if they were the physicians responsible for these patients for the duration of their hospitalization. Beds could only be included if both the patient and the resident agreed to participate. Each patient was paired with only one resident, but each resident included in the study could have one or more patients.

### Measurements

The patients completed sociodemographic questionnaires and the Hospital Anxiety and Depression Scale (HADS).^19^ HADS was scored using Brazilian validation data,^20^ which suggest that a cutoff of nine or over is predictive of depression (with a diagnostic sensitivity of 84.6% and a diagnostic specificity of 90.3%) or anxiety (with a diagnostic sensitivity of 93.7% and a diagnostic specificity of 72.6%).

The residents completed a sociodemographic and professional training questionnaire (specialization and number of years since graduation). For each participating patient in their care, they completed: (a) a version of HADS that had been modified to pose questions in the third person based on the resident's perception of the patient's emotional condition; (b) the Karnofsky Performance Status;^21,22^ and (c) the Difficulty in Helping the Patient (DTH) questionnaire.^23^ The DTH firstly asks open-ended questions about the patient's disease and the type of help that the patient requires. These questions are designed to trigger reflection about the patient and are not used in the analysis. Respondents are then asked a Likert-type question on the degree of difficulty involved in helping the patient (“To what degree do you find this patient difficult to help with the problems he/she presents?”) The response categories are: 0 = no difficulty; 1 = little difficulty; 2 = difficult; 3 = very difficult; and 4 = extremely difficult.

### Data collection and blinding

Four psychologists with prior experience in psychopathological assessments in general hospitals were trained to use the instruments described above. To maintain blinding, for each bed sampled, two psychologists were chosen at random: one to collect data from the patient and the other to collect data from the resident.

### Data analysis

The patient and resident characteristics were described in terms of frequencies and means. Differences relating to the DTH were examined in two ways: (1) patient and resident characteristics were compared using the T-test or ANOVA for independent samples; (2) the DTH score was dichotomized into “high” and “low” difficulty based on the mean value of participants' responses, and patient and resident characteristics were compared using the chi-square test. Taking the patient's self-reported HADS to be the gold standard for depressive or anxiety symptoms, the diagnostic agreement between the patient and resident responses to HADS was examined using the intraclass correlation coefficient (ICC) and kappa measurements. The Pearson correlation coefficient was applied to correlate the DTH score and Karnofsky index, and to correlate the DTH score and HADS score provided by the resident. Finally, the hypothesis that feelings of difficulty in helping patients can be driven as much or more by residents' perceptions of patients than by the patients' characteristics themselves was explored by means of a linear regression model. This was developed using the resident's DTH score as the dependent variable, and the patient's HADS score (reported by the patient and perceived by the physician) as independent variables. Other variables that were associated with the DTH score (with P < 0.1) could be added to the model as independent variables.

## RESULTS

Out of the total of 69 beds chosen, we included 56 in the study. Seven beds were excluded because the patients did not fulfill the inclusion criteria (one patient was a minor and six were under sedation), and another six were excluded because the resident responsible for them declined to complete the questionnaires. We thus carried out the study on 37 residents caring for 56 patients included (one resident for six patients, four residents for three patients each, six residents for two patients each, and 26 residents for one patient each) with 56 interviews with residents. The patients in the excluded beds did not differ significantly in age or gender from those included (P > 0.05).

Thirty-two patients (63.5%) were female. The patients' mean age was 51.4 years (standard deviation, SD = 16.8) and they had had a mean of 7.3 years of schooling (SD = 4.0). Fifty-five percent were married and 42.9% worked outside the home. The most prevalent admission diagnoses were circulatory diseases (26.5%), neoplasia (14.3%), osteomuscular diseases (16.3%) and urinary tract diseases (4.1%). The patients had a mean Karnofsky scale score of 67.8 (SD = 18.33), thus indicating, on average, that they were able to carry out activities of daily living but were unable to sustain normal levels of physical activity or do active work. The average duration of their illness was 24 months. About half (49%) had been previously hospitalized over the past year.

About two-thirds (71.4%) of the 37 residents were male, with a median age of 25 years. Only one of the 37 residents was in their first year of training and 67% were from the São Paulo area.

### Resident ratings of difficulty in helping patients

On average, the residents rated the patients as presenting relatively little difficulty. The mean rating on the DTH scale was 1.37 (SD = 0.94), between “little difficulty” and “difficult,” with normal distribution.

### Patient and physician ratings of anxiety and depression

According to their own ratings on HADS, 27.7% of the patients had depressive symptoms (HADS depression score of nine or over). The mean patient-reported HADS depression score was 5.77 (SD = 4.42). Thirty percent of the patients reported HADS anxiety scores of nine or over. The mean patient-reported HADS anxiety score was 7.0 (SD = 3.58).

The residents tended to rate a higher proportion of the patients as being depressed or anxious, in comparison with the ratings by the patients themselves. Using HADS, the residents rated 45% of patients as having some depressive symptoms (mean = 7.7; SD = 4.55), and 41.5% as having some anxiety symptoms (mean = 7.73; SD = 3.8).

The residents' and patients' reports on HADS showed low levels of agreement. The residents and patients were in agreement for 56.6% of the time regarding total depressive symptoms (ICC = 0.13; 95% confidence interval, CI = -0.66 to 0.54), and for 52.5% of the time regarding total anxiety symptoms (ICC = 0.22; 95% CI = -0.47 to 0.58). Put another way, the residents' sensitivity towards detecting depression (taking the patient rating to be the standard) was 50% (they identified six of the 12 patients who had rated themselves as depressed) and their specificity was 59% (out of 27 patients who were not depressed, residents correctly identified 16). The residents' sensitivity towards anxiety was 42% (correctly identifying five of the 12) and their specificity was 57% (correctly identifying 16 out of 28). There were only two HADS items for which the resident-patient agreement exceeded chance levels.

The residents' ratings of the patients' depression (HADS depression scores) correlated inversely with the patients' Karnofsky scores (r = -0.35; P = 0.031). Other than this, the residents' ratings of patient depression and anxiety did not show any correlations with patient gender, educational level, income, length of illness or length of hospitalization.

### Factors associated with residents' ratings of patient difficulty

The residents' ratings of difficulty in helping patients were greater when the residents were from areas of Brazil other than São Paulo (mean DTH score 1.93 when the residents were from other areas, versus 1.07 when the residents were from São Paulo; P = 0.04), but did not vary with other demographic characteristics of the residents (**Table 1**) or the patients' demographic and medical characteristics (**Table 2**).

**Table 1.** Distribution of the residents' degree of difficulty in helping patients due to the sociodemographic and professional characteristics of the residents

		Mean DTH score (SD)
Gender	Male	1.48 (0.97)
	Female	1.14 (0.86)
Age	Under 27 years	1.20 (0.83)
	28 years or over	1.41 (0.97)
Marital status	Without spouse	1.31 (0.83)
	With spouse	1.67 (1.51)
Place of work	Only at this institution	1.33 (0.96)
	Other jobs	1.45 (0.93)
Years after graduation	Up to one year	1.29 (0.83)
	One year or more	1.41 (1.01)
Specialty	None	1.00 (0)
	Clinical medicine	1.25 (0.90)
	Surgery	1.43 (0.98)
Daily hours	Up to 12 hours	1.36 (0.84)
	From 12 up to 24 hours	1.37 (1.01)
Weekly hours	Up to 72 hours/week	1.57 (0.98)
	More than 72 hours/week	1.15 (0.88)
City of origin*	From this city	1.07 (0.78)
	From other place	1.93 (1.0)
**Total**		**1.37 (0.94)**

* P < 0.05;

SD = standard deviation.

**Table 2.** Distribution of the residents' degree of difficulty in helping patients due to the sociodemographic and clinical characteristics of the patients

		Mean DTH score (SD)
Gender	Male	1.46 (1.13)
	Female	1.32 (0.86)
Age	18-42	1.17 (1.12)
	43-56	1.38 (0.74)
	≥ 57	1.36 (1.01)
Marital status	Without spouse	1.44 (1.24)
	With spouse	1.29 (0.90)
City of origin	From this city	1.41 (0.82)
	From other place	1.14 (1.46)
Income	Up to US$300 per month	1.36 (1.05)
	More than US$300 per month	1.24 (0.75)
Schooling	≤ 8 years	1.50 (1.00)
	> 8 years	1.08 (0.76)
Karnofsky index	90-80	0,92 (0.76)
	70-50	1.53 (0.84)
	40-20	1.63 (1.30)
Duration of illness	0-6 months	1.00 (0)
	7-24 months	1.25 (0.90)
	≥ 25 months	1.43 (0.98)
Previous hospitalizations	Yes	1.27 (0.92)
	No	1.38 (0.92)
Depression symptoms reported by the patient	Yes	1.42 (0.67)
	No	1.29 (1.01)
Anxiety symptoms reported by the patient	Yes	1.50 (0.80)
	No	1.25 (0.97)
**Total**		**1.37 (0.94)**

* P < 0.05;

SD = standard deviation.

The depression and anxiety symptoms perceived by the residents correlated with difficulty scores (r = 0.35 and P = 0.03; and r = 0.46 and P = 0.02, respectively) but depression and anxiety rated by the patients themselves did not (P > 0.05). The mean DTH score assigned to patients rated as depressed by the residents was 1.72 (SD = 1.02), compared with a mean of 1.09 (SD = 0.81) for those rated as not depressed. A similar difference was seen corresponding to the residents' ratings of patient anxiety: the mean DTH score for patients rated as anxious was 1.94 (SD = 0.90), compared with a mean of 0.96 (SD = 0.75) for those rated as not anxious. The residents who accurately rated their patients as depressed or anxious (i.e. their ratings of the patient using HADS agreed with that patient's own report) were no more likely to consider the patient difficult to help than were residents who made inaccurate ratings of the patient's mental health (**Table 3**).

**Table 3.** Distribution of the physicians' degree of difficulty in helping patients due to the professionals' perceptions regarding the patients' symptoms and correct or incorrect identification of the patients' symptoms

		Mean DTH score (SD)
Perception of depressive symptoms	Yes*	1.72 (1.02)
	No	1.09 (0.81)
	Correct perception	1.14 (0.89)
	Error	1.59 (0.94)
Perception of anxiety symptoms	Yes†	1.94 (0.90)
	No	1.96 (0.75)
	Correct perception	1.14 (0.85)
	Error	1.53 (0.96)

* P < 0.05;

† P < 0.01;

SD = standard deviation.

We performed two linear regression analyses to examine predictors of residents' difficulty in helping: one for residents who labeled the patient as anxious and one for residents who labeled the patient as depressed (**Tables 4** **and** **5**). In both, the dependent variable was the residents' DTH score. We also included, as independent variables, the patients' own rating of anxiety, their Karnofsky index and their educational level, and whether the resident came from a city other than São Paulo. Labeling a patient as presenting anxiety was associated with a nearly one point increase in difficulty rating (0.94 points; 95% confidence limits 0.48-1.40). Residents who were not from São Paulo rated patients, on average, as 0.67 points more difficult (95% confidence limits 0.18-1.16). The patients' self-rated anxiety score had a borderline significant relationship with the residents' ratings of difficulty: the difficulty tended to increase as the patient's self-rated HADS score increased. The patient's Karnofsky index and educational level were not related to difficulty. This model explained 48% of the variation in DTH score.

**Table 4.** Physician-perceived and patient-reported anxiety as predictors of difficulty

Variable	Coefficient	Significance	95% confidence interval	
			Minimum	Maximum
Resident-perceived anxiety (HADS ≥ 9)	0.94	< 0.001	0.48	10.40
Resident's city of origin	0.67	0.009	0.18	10.16
Patient-reported anxiety (HADS ≥ 9)	0.07	0.053	0	0.14
Karnofsky index	- 0.003	0.58	- 0.02	0.01
Patient's educational level	- 0.25	0.30	- 0.74	0.23
Constant	0.11	0.87	- 10.2	10.5
Adjusted R^2^ = 0.48				

HADS = Hospital Anxiety and Depression Scale

**Table 5.** Physician-perceived and patient-reported depression as predictors of difficulty

Variable	Coefficient	Significance	95% confidence interval	
			Minimum	Maximum
Resident-perceived depression (HADS ≥ 9)	0.59	0.038	0.04	10.14
Resident's city of origin	0.96	0.002	0.37	10.55
Patient-reported depression (HADS ≥ 9)	- 0.023	0.46	- 0.09	0.04
Karnofsky index	- 0.01	0.20	- 0.028	0.006
Patient's educational level	- 0.22	0.46	- 0.83	0.38
Constant	0.99	0.23	- 0.66	20.63
Adjusted R^2^ = 0.26				

HADS = Hospital Anxiety and Depression Scale.

The regression looking at resident and patient ratings of depression yielded similar results, although the impact on difficulty scores was of smaller magnitude. On average, patients labeled as depressed by the residents had difficulty scores that were 0.59 points greater (95% CI 0.035-1.14), but there was no indication of a relationship between the patient's self-rated HADS depression score and difficulty. In both the anxiety and depression regressions, we checked for and found no evidence of an interaction between residents' ratings of patient mood and the resident's city of origin.

## DISCUSSION

We found that the residents' perceptions of their patients' anxiety and depression predicted difficulty ratings independently of the patients' objectively measured medical or psychiatric status. As in another study on generalist residents, agreement with an independent measurement of patient mental health was only slightly better than chance.^24^ There was an indication that patients' self-rated anxiety symptoms might also be related to difficulty, but no indication of a relationship with self-rated depression. Other measurements, on patients' socioeconomic status, functional status and length of hospitalization did not correlate with residents perceiving them as difficult to help.

Truly anxious patients may be seen as more demanding because they ask more questions, have more physical concerns that need to be addressed, or simply seem to require more of the doctor's presence at their bedside.^2,12,15^ Depressed patients can be irritable and also seem demanding, but they may also be apathetic and passive. In cases of short hospitalization, residents might even perceive this second group of patients with depression as easier to care for. Residents may not perceive depression as something for which they are responsible, while they may feel obligated to respond to an anxious patient's desire for reassurance with further medical evaluation. In outpatient settings, physicians frequently make distinctions between mental health problems for which they feel responsible and those that they feel should be referred.^4^

We do not know why the residents found any particular patient to be anxious or depressed when the patient himself or herself did not. The patients rated as anxious or depressed by the residents tended to have poorer levels of function (as indicated by lower Karnofsky index), but otherwise did not seem distinct from the patients who were not perceived by the residents as having mood problems. It is possible, however, that the residents were noticing patient characteristics that were not detected when the patients completed the HADS questionnaire themselves. The patients may have displayed different emotions when the resident was present, compared with when the study psychologist met them to complete the HADS. This may have been because the resident had come to administer a medical procedure or because, when the resident was present, the patients feared receiving unwelcome news about their condition. Another explanation could be that the residents were themselves anxious at the time of their encounters with patients and projected that emotion onto the patient. In one study on family practice residents, anxiety about death was correlated with relative intolerance of ambiguity and feeling anxious about close relationships.^25^

Regarding the association of difficulty with physicians coming from other cities, we were unable to find other studies relating to this topic. This indicates the importance of deepening the investigation in relation to the living conditions of professionals, particularly recent graduates.^9-11^ One possible explanation is that when residents are newly-graduated professionals, generally aged around 24, and they move to another city and take on new responsibilities in an unfamiliar university hospital environment, they have less social support of their own, given their distance from home and the potential difficulty of adapting to a new living environment. This may reduce their threshold for feeling strained by the demands of work in general, or in relation to particular patients.

Finally, our results provide more data to support the need for interventions regarding residents' difficulty in helping patients. If residents' discomfort with patients has been driven by patients' actual mental health state, the interventions might include providing more training on management of comorbid mental health problems, or providing better access to psychosocial support for those patients. However, in our study, the discomfort was related more to residents' perceptions of the patients as having mental health problems (regardless of whether this was true). Thus, interventions may need to focus more on the processes of communication and diagnosis,^3^ and on achieving better understanding of the cues (coming from patients or from the residents' past experience) that result in misperception of patients' emotional state.^2,15^

### Limitations

The results from this study should be interpreted in context. They are based on reports from a single group of residents who found that their patients, as a group, were relatively easy to care for. There may have been some degree of social acceptability bias in the residents' ratings of difficulty, thereby lowering these ratings across all patients. However, this would tend to make our results conservative, in that we would expect it to weaken the relationship of difficulty with perceived patient mental health problems.

## CONCLUSIONS

The frequency with which residents find it difficult to treat patients with emotional problems may be increased by the residents' inability to identify which patients are truly experiencing depression or anxiety. Finding mechanisms to help residents with mental health diagnosis may be helpful. Feelings of difficulty among residents appear to be independently related to sociodemographic and clinical differences with patients. This suggests that training and consultation around broader assessment of patients' psychosocial status could contribute towards smoother interactions between residents and their patients.
